# Are Silver and Gold Nanoparticles Obtained by ‘Green’ Synthesis Biocompatible?

**DOI:** 10.3390/nano16160993

**Published:** 2026-08-12

**Authors:** Lucas Reijnders

**Affiliations:** Faculty of Science, Institute for Biodiversity and Ecosystem Dynamics, Science Park 904, 1090GE Amsterdam, The Netherlands; l.reijnders@uva.nl

**Keywords:** ‘green’ synthesis, biosynthesis, gold nanoparticles, silver nanoparticles, biocompatible

## Abstract

In scientific literature, the biosynthesis of gold and silver nanoparticles and synthesis of these nanoparticles using small organic molecules such as citrate have been called ‘green’ processes. In abstracts of scientific publications, gold or silver nanoparticles obtained by ‘green’ synthesis have rather frequently been characterized as biocompatible. Biocompatible means having no negative impact on exposed organisms. Two reasons have been used to underpin this characterization. The first is the biocompatibility of the substances used in ‘green’ synthesis and coating nanoparticles. This reason lacks a solid empirical basis. The second reason given for biocompatibility is limited testing of the nanoparticles obtained by ‘green’ synthesis (mainly in vitro testing). Such limited testing is inconclusive. Use-specific comprehensive in vitro and in vivo testing, including clinical studies, and control of hazardous substances such as lipopolysaccharide and flagellins are needed to provide a solid basis for their characterization as biocompatible for humans.

## 1. Introduction

Gold and silver nanoparticles can be synthesized by using small organic molecules such as citrate or by biosynthesis using a variety of organisms and substances derived thereof. This is often described in terms of ‘green nanoparticle synthesis’ [1]. In scientific publications, gold and silver nanoparticles obtained by ‘green’ synthesis have rather frequently been characterized as biocompatible. For instance, according to a search on 3 July 2026, the abstracts of 240 publications in the Core Collection of the Web of Science used the characterization ‘biocompatible’ for silver particles obtained by green synthesis, whereas the corresponding number for gold nanoparticles was 130. Biocompatible means having no negative impact on exposed organisms [2]. Gold and silver nanoparticles synthesized using amino acids, ascorbate, citrate and glucose have been described as biocompatible [3,4,5,6,7,8,9,10,11], but the characterization ‘biocompatible’ most often refers to biosynthesized Ag and Au nanoparticles. Plant derivatives such as extracts are most often used in scientific studies dealing with the biosynthesis of Ag and Au nanoparticles [12,13,14,15,16]. This paper will evaluate for the first time the correctness of biocompatibility claims regarding silver and gold nanoparticles obtained by ‘green’ synthesis.

In Section 2 a brief description is given of the method used in preparing this manuscript. Section 3 considers whether silver and gold nanoparticles obtained by ‘green’ synthesis are biocompatible. Section 4 addresses the therapeutic application of biosynthesized gold and silver nanoparticles in human medicine. Section 5 will present the conclusions of this paper.

## 2. Methods

Databases of major publishers of scientific literature in the field of nanomaterials (ACS, Elsevier, Frontiers, IOP Publishing, MDPI, RSC, Sage, Springer-Nature, Taylor and Francis, Wiley), the Web of Science core collection and Google Scholar were searched for publications since 2010 regarding the subjects raised in this paper.

## 3. Biocompatibility of Gold and Silver Nanoparticles Obtained by ‘Green’ Synthesis

In scientific papers, two main approaches could be found supporting the characterization of gold and silver nanoparticles obtained by ‘green’ synthesis as ‘biocompatible’. The first approach links the ‘biocompatibility’ of Ag and Au nanoparticles obtained by ‘green’ synthesis to biosynthesis or the substances used in the synthesis, often with emphasis on those substances present in nanoparticle coatings, also called cappings. The second approach supports the characterization as ‘biocompatible’ by testing the synthesized nanoparticles. Section 3.1 details the first approach. Section 3.2 addresses the second.

### 3.1. Biocompatibility Linked to the Substances Used in ‘Green’ Synthesis or Biosynthesis

Table 1 presents examples from the scientific literature since 2010 linking biocompatibility to biosynthesis or substances used in ‘green’ synthesis. The variety of reasons given for biocompatibility is emphasized in the table, while retaining the importance of plant-based extracts.

Regarding the question of whether the substances present during specifically ‘green’ biosynthesis of Ag and Au nanoparticles confer biocompatibility, there is, firstly, the matter of whether the substances used in biosynthesis are indeed biocompatible. Focussing on plants, derivatives of which are most often used in ‘green’ synthesis and also prominent in Table 1, secondary metabolites such as flavonoids, saponins, alkaloids, polyphenols, tannins, and terpenoids may be present in nanoparticle cappings [47,48,49,50,51,52,53,54,55,56,57,58,59,60,61]. Beneficial impacts of these compounds have been stressed (e.g., refs. [51,62,63,64,65,66]), but such compounds may also be problematic.

Gold and silver nanoparticles can be synthesized with flavonoids as reductants and may be capped by flavonoids (e.g., refs. [67,68,69,70,71]). Silver nanoparticles synthesized in this way are considered for infection control for humans (e.g., refs. [69,70,72]). Flavonoids are estrogens [73]. In view of the potential negative impacts of additional exposure to estrogens for substantial parts of the human population [74,75], their presence in coatings of silver nanoparticles used for infection control would seem problematic. Furthermore, several flavonoids have shown hepatotoxicity, neurotoxicity and nephrotoxicity [71,76].

Some saponins have been found to negatively affect the hematological system, liver and kidneys [76]. In this context it may be noted that Abdelaziz et al. synthesized silver nanoparticles using saponins [77]. Oral administration of these saponin-synthesized silver nanoparticles to pregnant rats negatively affected maternal. and fetal renal and hepatic tissues [77]. There are also concerns as to a number of alkaloids regarding neurotoxicity, hepatoxicity, cardiotoxicity and nephrotoxicity [78,79]. Various polyphenols have shown developmental toxicity, interference with the thyroid and genotoxicity [80]. Several tannins have been found to be genotoxic, carcinogenic and hepatotoxic [53]. It may be noted that Assar et al. [81] showed that silver nanoparticles synthesized with citrate and tannins, when applied intraperitonially, can be hepatoxic in rats. A number of terpenoids have been shown to be immunotoxic, genotoxic, carcinogenic, hepatotoxic and cardiotoxic [82,83,84].

In fungal synthesis, mycotoxins may be a matter of concern. Varieties of fungi reportedly used in the laboratory-scale synthesis of gold and silver nanoparticles such as *Fusarium acuminatum*, *Fusarium oxysporum*, *Fusarium solani*, *Aspergillus fischeri*, *Aspergillus flavus*, *Aspergillus fumigatus* and *Aspergillus niger* [31,32,85,86] may generate mycotoxins [87,88]. Mohammadjani et al. [89] used cell-free extract of the fungus *Sarocladium subulatum* to synthesize Ag nanoparticles and found that the extract contained a variety of hazardous substances, including potentially immunotoxic substances, potential carcinogens and mutagens.

There are furthermore bacteria-derived immunity-modulating substances that may negatively impact the quality of nanomedicines, including flagellins (proteins involved in bacterial motility) and lipopolysaccharide (endotoxin) [90]. Lipopolysaccharide originates in the cell walls of Gram-negative bacteria and may bind to the surfaces of Ag and Au nanoparticles during nanoparticle synthesis and processing [91,92,93,94,95]. In vitro experiments found that lipopolysaccharide bound to gold nanoparticles may induce inflammatory effects and may interfere with corona formation [96].

Coatings resulting from biological synthesis may furthermore contain non-self proteins, originating in the organisms that serve biosynthesis [19,27,54,89,97,98,99,100,101,102,103,104]. Non-self proteins may trigger adverse immunological reactions and cytotoxicity [61,94,101,105,106,107,108]. Focussing on plants, derivatives of which are most often used in ‘green’ synthesis, it may be noted that allergy-like reactions to herbal drugs and food plants linked to proteins have been reported [108,109]. The impact of non-self proteins after binding to gold and silver nanoparticles is uncertain, because non-self proteins undergo conformational changes when strongly adsorbed to nanoparticles [110,111]. Furthermore, coronas with self-proteins are formed following exposure, and the composition of the combined self- and non-self protein coronas may undergo changes on migration through the body [94,112,113]. Knowledge about the interactions of organisms and metal nanoparticles with cappings containing non-self proteins that are relevant to biocompatibility is limited [101,107].

Secondly, it can be noted that the use of biological materials in the synthesis of nanoparticles does not necessarily preclude adverse effects that appear to be at variance with biocompatibility. When extracts from plants or microalgae are applied in nanoparticle biosynthesis, adverse effects may occur. Aravinthan et al. [114] investigated oral administration of gold nanoparticles synthesized with *Helianthus tuberosa* extract to rats. Induction of hypoglycemia and increase in LDL-cholesterol levels were shown, and chronic administration was linked to lung tissue damage [114]. Akkam et al. [115] studied intraperitoneal injections of gold nanoparticles synthesized using leaf extract of *Ziziphus* in mice and found adverse effects, particularly in the kidneys. Tareq et al. [116] investigated the oral administration of silver nanoparticles synthesized using extract of *Psidium guajava* to rats. Dose-dependent negative impacts on neurotransmitters in the brain were shown [116]. Opris et al. [117] noted dose-dependent neurobehavioral changes and damage to brain cells, including neurons, after oral administration to rats of silver nanoparticles synthesized with *Cornus mas* extract. Tarbali et al. [118] showed negative effects of intraperitoneally injected silver nano particles synthesized using *Myrthus communis* on the behaviour and memory of rats. Silver nanoparticles synthesized with extract of the microalga *Chlorella vulgaris* were shown to be more toxic to chicken embryos than to *Salmonella enterica* [119]. Botteon et al. [71] synthesized gold nanoparticles using Brazilian red propolis extract. Propolis is a plant-derived material generated by bees for which health benefits have been claimed [120]. Botteon et al. [71] found developmental toxicity of these gold particles on zebrafish embryos.

As to the use of fungi in the biosynthesis of gold and silver nanoparticles, Fatima et al. [121] reported the dose-dependent cytotoxicity (reduction in cell viability) to cell lines J774 (mouse macrophage) and THP1a (human macrophage) on exposure to silver nanoparticles synthesized by *Bipolaris tetramera*. Pourali et al. [122] and Yahyaei et al. [123] found cytotoxicity of gold nanoparticles synthesized using *Fusarium oxysporum* to human fibroblast cell line CIRC-MFL and mouse fibroblast cell line NIH3T3, respectively. Although Salem [102] stated that silver nanoparticles extracellularly synthesized with *Saccharomyces cerevisiae* showed promise as safe antibacterial agents, Skóra et al. [124] reported substantial dose-dependent reduction in the metabolic activity of human keratinocytes (HaCaT -CRL2523) and mouse embryonic fibroblasts (NIH3T3) by Ag nanoparticles extracellularly synthesized using *Saccharomyces cerevisiae.* Zawadzka et al. [125] found substantial haemolytic effects and cytotoxicity of silver nanoparticles synthesized by using filtrate of the fungus *Gloeophyllum striatum*, which they considered at variance with applications in human medicine.

Regarding the use of bacterial substances in ‘green’ synthesis, Vijayakumar et al. [126] reported a substantial dose-dependent negative impact on the viability of mouse embryonic fibroblast (3T3) cells by silver nanoparticles synthesized using the supernatant of pro-biotic bacteria. This is at variance with the paper by Algburi et al. [45] (see Table 1). Zhang et al. [127] reported toxicity to spermatoginial stem cells and somatic cells involved in the reproductive systems of male mice on exposure to Ag nanoparticles synthesized with substances derived from *Bacillus cereus*.

To sum up, not all appears to be necessarily well with the biocompatibility of Ag and Au nanoparticles obtained by biosynthesis. In view of this there is a strong case for empirical testing of the actual biocompatibility of such nanoparticles. Testing biocompatibility is the subject of Section 3.2. Furthermore, there is no reason to doubt the biocompatibility for mammals of amino acids mentioned in Table 1 [3], and the same holds for other small organic molecules such as ascorbate, citrate and glucose. But whether a coating of biocompatible small organic molecules confers biocompatibility would rather seem a matter for proper testing, which is discussed in Section 3.2. Table 2 presents data of testing Ag and Au nanoparticles synthesized (and coated) with amino acids, ascorbate, citrate and glucose regarding their impact on cell lines.

### 3.2. Showing Biocompatibility by Testing Ag and Au Nanoparticles Obtained by ‘Green’ Synthesis

Characterization as ‘biocompatible’ can be supported by testing the synthesized nanoparticles. Tests may be in vitro (Section 3.2.1) or in vivo (Section 3.2.2). A blanket-claim of biocompatibility would imply that all organisms and all potential negative impacts are covered by testing. Comprehensive testing covering all organisms and all potential negative impacts would seem in practice not feasible, as impacts of nanoparticles can be species-, tissue- and organ-dependent, and can also vary with the type of exposure [2]. Comprehensive testing of the potential negative impacts of specific uses in human or veterinary medicine would, however, seem feasible [128,129], though not easy. Ag nanoparticles, including their coronas, are subject to complex transformations in the mammalian body [113,130]. The fate of Au nanoparticles in the human body can substantially vary between individuals, depending on endothelial fenestral pore sizes, age and genetics [131]. Coronas of Au nanoparticles may undergo changes on migration through the body [113], and the distribution of gold nanoparticles in the body is difficult to manipulate [132].

In this section, tests will be considered that can be relevant to biocompatibility regarding use in human medicine. In practice, only one or a few of such tests could be found in the scientific literature regarding gold or silver nanoparticles obtained by a specific ‘green synthesis’ (e.g., using a specific leaf extract from a specific plant). An exception involves citrate-coated silver nanoparticles, for which a substantial set of tests that can be relevant to potential applications in human medicine is available. It should be noted, though, that particle size and shape and the agglomeration of the citrate-coated silver particles in the tests varied, which is known to have an impact [133], and the correspondence between the tests and the conditions in actual medical applications was not established.

Almeida et al. [7] found that, depending on the mass-based concentration of silver nanoparticles synthesized and coated with citrate, the cell viability of mouse embryonic fibroblast NIH3T3 decreased in a time-dependent way to about 60% of the cells present in the test after 72 h in the presence of serum. This is in line with the paper by Barbalinardo et al. [134], who found that citrate-capped silver nanoparticles are cytotoxic in a dose- and time-dependent way when internalized by mouse embryonic fibroblasts (NIH3T3) and that internalization is mediated by the presence of a protein corona obtained from the serum. Mohammadi and Amini [135] reported on citrate-coated Ag nanoparticles in a large mass-based concentration-dependent reduction in human dermal fibroblast cells in the presence of serum. There is empirical evidence that citrate-coated Ag nanoparticles can be neurotoxic in vivo in mice and rats following oral exposure and can have negative impacts on human embryonic stem-cell-derived neural progenitors during neuronal differentiation and on human embryonic stem-cell-derived glutamatergic neurons [136,137,138,139]. Danila et al. [140] reported that oral administration of citrate-coated silver nanoparticles to pregnant Wistar rats caused neurotoxicity in offspring. Citrate-coated silver nanoparticles can accumulate in, and may cause damage to, the liver [141]. Scoville et al. [142] showed that citrate-coated Ag nanoparticles induced acute lung inflammation in mice following inhalation. Cardiovascular homeostasis and electrophysiology in mice can be negatively impacted by inhaled citrate-coated silver nanoparticles [143,144]. Citrate-coated Ag nanoparticles were shown to be immunotoxic in vitro (using human peripheral blood mononuclear cells from healthy donors) with differences between the sexes and ages [145]. Also, negative impacts of citrate-synthesized silver nanoparticles on human lymphocyte activation in vitro have been reported [146]. As for human fibroblasts, de Araujo Viera et al. [147] have shown that (in the absence of impact on cell viability) in vitro their function can be impaired by citrate-coated Ag nanoparticles. Citrate-coated Ag nanoparticles have been shown to be more genotoxic in standard in vitro assays than similar-sized Ag nanoparticles with a capping of polyvinylpyrrolidone [148]. In vivo studies in mammals found genotoxicity of citrate-coated Ag nanoparticles [149]. A study in which citrate-coated silver nanoparticles were painted on ARPE-19 cells (human retinal pigmented epithelial cells) showed changes indicating damage to DNA [150]. Taken together, these empirical findings regarding citrate-coated silver nanoparticles do not exclude biocompatibility for all citrate-coated silver nanoparticles. However, the tests discussed in this paragraph do not support the characterization of biocompatibility for the tested citrate-coated silver nanoparticles.

#### 3.2.1. In Vitro Tests

In scientific studies stating that Ag and Au nanoparticles obtained by ‘green’ synthesis are biocompatible, the supporting tests are often in vitro. The number of them may vary. Such tests may use human blood cells to establish haemolysis (e.g., refs. [151,152,153,154,155,156,157]). There is furthermore biocompatibility testing using one or more human or mammalian cell lines. For biocompatibility testing, the use of normal cell lines would seem to be the proper choice. The normal cell lines used in biocompatibility testing vary. Examples of such cell lines used in tests claiming to show ‘biocompatibility’ are presented in Table 2. When the studies referred to in Table 2 address therapeutic impacts on cancer, cell lines of cancerous cells are also tested, with the results often showing that the negative impacts on normal cell lines are lower [62,158,159,160], though the opposite may also occur (e.g., ref. [124]).

**Table 2:** Normal cell lines used for in vitro testing of gold and silver nanoparticles, obtained by ‘green’ synthesis, claimed to show ‘biocompatibility’.

Cell Lines Used for In Vitro Tests to Support the Characterization ‘Biocompatible’	Effects	Type of Biosynthesized Nanoparticle	Substances Involved	References
Human lung epithelial A549 cells	Cell viability	Ag	Garlic clove extract	[161]
Mouse fibroblast cells L929	Cell viability	Ag	*Ammania baccifera* extract	[162]
Human dermal fibroblast	Cell viability	Au	*Origanum vulgare* extract	[163]
		Ag	Apigenin (flavonoid)	[135]
Fibroblast L 929 cells	Cell viability	Au	Cucurmin	[164]
		Ag	*Lantana Montevidensis*	[165]
Mammalian HEK 293 embryonic kidney cells	Cell viability	Ag	Extract from mixture *Asafoetida* and *Agave americana* leaf	[166]
Human HEK-293 embryonic kidney cells	Cell viability	Ag, Au	*Gelidium pussilum* extract,	[158]
			*Olea europaea* leaf extract,	[167]
			Amino acids,	[10]
			*Anabaena variabilis* extract	[159]
Human fibroblast normal cells	Cytotoxicity at low concentrations	Au	*Ziziphus nummularia* extract	[62]
Human dermal fibroblast cells	Cell viability	Au	Amino acids	[4]
Human gingival fibroblast cells	Cell viability, cell morphology	Ag	Glucose	[5]
Human epithelial cell line HEK 293T	Cell viability	Ag	L-cysteine and citrate	[6]
Human mesenchymal stem cells	Cell viability	Ag	*Olea europaea* extract	[167]
Mouse embryonic fibroblasts NIH3T3	Cell viability	Ag	Citrate	[7]
L 929 murine fibroblasts	Cell viability	Au	Alginates	[168]
		Ag	*Ipomoea cairica* leaf extract	[169]
Mouse macrophage cells RAW 264.7	Cell viability	Au	Citrate	[8]
			Ascorbic acid	[11]
			*Lilium wallichianum* leaf extract	[170]
Human HEK kidney cells	Cell viability	Ag	*Cyperus rotundus*	[171]
Mouse embryonic fibroblast NIH3T3	Cell viability, cell morphology	Ag	Garlic extract	[172]
		Au	Shark Chondroitin sulfate	[160]
		Ag	Green tea extract	[173]
Monkey kidney cells VERO	Cell viability, genotoxicity	Ag	Ascorbic acid	[9]

There is no uniformity in testing for haemolytic impact [154] and there is no uniformity as to the criterion for impacts on human blood cells that can be considered biocompatible. Shanmugam et al. [174] characterized Ag nanoparticles synthesized with *Malus pumila* extract as biocompatible while finding a dose-dependent haemolysis of up to 11.6%. Whereas Foo et al. [152], Das et al. [153] and Talukder et al. [175] considered a haemolysis of <10% of blood cells biocompatible, Gul et al. [155], Sateesh et al. [176] Nejati et al. [177] and Manzoor et al. [157] used a value of <5% and Kumar et al. [151] a value of <1% for biocompatible haemolysis. As to cell viability, which emerges in Table 2 as the effect most often studied, concentration ranges tested vary and actual criteria for biocompatibility vary too. In the studies considered in Table 2, criteria used vary from ‘>60% of cells viable’ to ‘no (significant) impact on cell viability’. Moreover, the use of different cell lines for cell viability tests leads to scattered outcomes [2]. Testing cell viability would furthermore seem to be a way of showing biocompatibility that leaves out much. Negative impacts by Ag and Au nanoparticles obtained by ‘green’ synthesis relevant to biocompatibility may occur at concentrations below those that impact cell viability. Cases in point involve immunotoxicity, genotoxicity, inflammation and negative impacts on functionality of organs [2,143,144,145,147,148,178,179,180,181].

More in general, the outcomes of testing haemolytic impact and/or responses of one or two cell lines to exposures by nanoparticles may well be at variance with in vivo effects, as many interactions between nanoparticles and the body relevant to biocompatibility remain unexplored in such in vitro testing [2,128,131,154,182,183,184]. In view of this, studies testing haemolytic impact and impacts on the viability of normal cell lines do not provide a firm basis on their own for the characterization of ‘biocompatible’.

#### 3.2.2. In Vivo Tests

In vivo testing has been presented to support ‘biocompatibility’, occasionally in addition to in vitro testing. Here it is explained what is tested, while Table 3 outlines the matters relevant to biocompatibility not clarified or covered by the tests. Baskaran et al. [185] tested the biocompatibility of biosynthesized silver nanoparticles on the basis of acute death and (unspecified) ‘toxic signs’ in Wistar albino adult female rats upon oral exposure. To establish biocompatibility, Mahmud et al. [186] and Hossain et al. [187] tested kidney and liver toxicity after intravenous administration of silver nanoparticles synthesized, and respectively coated, with plant-derived substances in rats. Aljohani et al. [188] tested acute toxicity after intraperitoneal injection of rats with gold nanoparticles synthesized using Egyptian propolis extract. Stalin et al. [170], who also tested cell viability using mouse macrophages (see Table 2), studied acute toxicity to brine shrimps (*Artemia salina nauplii*) to establish the biocompatibility of biosynthesized gold nanoparticles intended for human medicine. Both Aljohani et al. [188] and Stalin et al. [170] concluded that the nanoparticles could be safely used. Kahn et al. [189] tested the biocompatibility of biosynthesized gold nanoparticles intended for application against cancer by studying survival and changes in morphology of zebrafish (*Danio rerio*) larvae. Jaswanthika et al. [190] tested the biocompatibility of biosynthesized silver nanoparticles for application against bacteria causing cellulitis by studying the survival of zebrafish (*Danio rerio*) embryos and found substantially reduced survival at 25 µg/L, increasing during 96 h of exposures. The 25 µg/L concentration was the same as or was less than the minimum inhibitory concentrations for several tested bacterial strains. Jaswanthika et al. [190] nevertheless used the characterization ‘biocompatible’.

In view of the limited coverage of negative impacts shown in Table 3 and uncertainties about extrapolation to human medicine [191,192,193], the in vivo studies discussed in this section do not provide a firm basis for the characterization of ‘biocompatible’ in human medicine.

#### 3.2.3. Comprehensive Testing Protocols

Whether Ag or Au nanoparticles synthesized in a ‘green’ way are indeed biocompatible should be established by the application of a comprehensive testing protocol covering the potential negative impacts for intended uses involving both in vitro and in vivo empirical testing (see also refs. [2,52,112,128,194,195,196]). Standard 10993 of the International Standards Organization (ISO-10993) [197] provides guidance regarding the testing of nanomaterials for use in human medicine, covering both in vitro and in vivo testing, including clinical testing. This standard covers such matters as sensitizing/immunotoxicology, chronic toxicity, neurotoxicity, genotoxicity and reproductive/developmental toxicity [194,196]. As biocompatibility testing evolves, there is a case for including recent developments in comprehensive testing protocols. Recent developments in the comprehensive testing of nanomaterials for use in human medicine have been reviewed [198]. Additionally, there is guidance on the testing of specific impacts of nanoparticles, such as hepatotoxicity [183], immunotoxicty [90] and genotoxicity [179]. Furthermore, there is a case for measuring and controlling hazardous contaminants that may be present in nanomedicines [90]. No scientific publication has been found that applies a testing protocol for biocompatibility as outlined in ISO-10993 or another comprehensive test protocol to Ag or Au nanoparticles synthesized in a ‘green’ way for use in human medicine. Manzoor et al. [157], characterizing the Ag nanoparticles obtained by biosynthesis as biocompatible, stress in the title of their paper ‘a comprehensive biological evaluation’, but the only biocompatibility test presented regards haemolytic impact.

## 4. Therapeutic Application of Biosynthesized Ag and Au Nanoparticles in Human Medicine

Comprehensive testing for biocompatibility, including testing for the presence of hazardous substances, is a requirement for registration as a therapeutic medicine in the European Union and the United States of America [2,90,128,198,199]. A recent set of hazardous substances for which testing is required by the US Food and Drug Administration for registration as nanomedicine involves five innate immune modulating substances, including lipopolysaccharide, ß-glucan and flagellin [90]. Halley and Dobrovolskaia [90] have argued that the list of hazardous substances should be extended. When use against (multi-)antibiotic-resistant pathogens is intended, there is a case for addressing concerns about the emerging resistance of microbes against silver nanoparticles [200,201,202].

As promising data about in vitro effects of nanoparticles can fail to translate to in vivo efficacy [184], there is the need for additional in vivo efficacy studies, including clinical trials. Commercial production and registration-based use of biosynthesized gold and silver nanoparticles would require that production runs lead to reproducible results (e.g., refs. [202,203,204,205,206]. In the case of synthesis using plant extracts, variations in plant material and extractions and slight changes in syntheses can lead to substantial variations in the nanoparticulate product outputs (e.g., refs. [204,207,208]). Currently, in nanoparticle biosynthesis, the reproducibility of size, morphology, coating and functionality is problematical [41,204,206,209,210]. I have not been able to identify the registration of biosynthesized Ag and Au nanoparticles for use in therapeutic medicine by the European Medicines Agency [211] or by the US Food and Drug Administration [212] (also refs. [184,194,198,199,205,213]).

## 5. Conclusions

The characterizations in scientific papers of gold and silver nanoparticles generated by ‘green’ synthesis as ‘biocompatible’ discussed here lack a solid basis of use-specific comprehensive in vitro and in vivo testing. Use-specific comprehensive in vitro and in vivo testing and control of hazardous substances such as endotoxins are needed to provide a solid basis for biocompatibility claims. No published scientific study was found which shows the application of a comprehensive testing protocol, such as ISO-10993, to gold or silver nanoparticles synthesized in a ‘green’ way. No registration of biosynthesized Ag and Au nanoparticles for use in medicine by the European Union (European Medicines Agency) or by the US Food and Drug Administration (FDA) could be identified.

## Figures and Tables

**Table 1:** Reasons given for the ‘biocompatibility’ of Ag and Au nanoparticles obtained by ‘green’ synthesis.

Reason Given for Biocompatibility	Element(s) in Nanoparticle	References
Aqueous extracts of earthworm and snail used in synthesis contain valuable biological entities	Ag, Au	[17]
Bio-directed synthesis	Ag	[18]
Capping with biomolecules of natural origin	Ag	[19]
Green (bio)synthesis	Ag	[20,21,22]
Natural biocorona	Au	[23]
Production by biological systems	Au	[24]
Synthesis/capping with citrate and conjugation with bovine serum albumin	Au	[25]
Synthesis and capping by non-toxic sodium lignosulfonate	Ag	[26]
Use of algal extracts in synthesis	Ag	[27]
	Au	[28]
	Ag	[29]
Use of amino acids in synthesis	Ag	[3]
Use of bio-reducing and bio-capping agents	Ag	[16]
Use of carbohydrates	Au	[30]
Use of fungi in synthesis, leading to capping with fungal constituents;	Ag, Au	[31,32]
Use of herbal drug extract in synthesis	Ag, Au	[33]
Use of plant extract in synthesis	Ag, Au	[14,29,34,35,36,37,38,39,40,41,42,43,44]
Use of probiotic substances	Ag	[45]
Use of specified mushroom extracts	Au	[46]

**Table 3:** Matters relevant to biocompatibility not considered by references in which biocompatibility is claimed.

Reference	Matters Relevant to Biocompatibility Not Covered or Clarified by the Reference
Baskaran et al. [185]	Immunotoxicity, reproductive toxicity and genotoxicity.
Aljohani et al. [188]	Chronic toxicity, immunotoxicity, genotoxicity, or developmental toxicity.
Mahmud et al. [186]; Hossaini et al. [187]	Neurotoxicity, immunotoxicity, developmental toxicity and genotoxicity.
Kahn et al. [189]	Immunotoxicity and neurotoxicity.
Stalin et al. [170]	Chronic toxicity, immunotoxicity, genotoxicity, and developmental toxicity.
Jaswanthika et al. [190]	The concentration tested 25 µg/L was the same as, or was less than, the minimum inhibitory concentrations for several tested bacterial strains.

## Data Availability

No new data were created or analyzed in this study. Data sharing is not applicable to this article.
